# Prognosis and Genomic Landscape of Liver Metastasis in Patients With Breast Cancer

**DOI:** 10.3389/fonc.2021.588136

**Published:** 2021-03-11

**Authors:** Chonglin Tian, Sujing Liu, Yongsheng Wang, Xianrang Song

**Affiliations:** ^1^Graduate School, Shandong First Medical University and Shandong Academy of Medical Sciences, Jinan, China; ^2^Shandong Cancer Hospital and Institute, Shandong First Medical University and Shandong Academy of Medical Sciences, Jinan, China; ^3^Department of Radiation Oncology, The Affiliated Yantai Yuhuangding Hospital of Qingdao University, Yantai, China

**Keywords:** breast cancer, liver metastasis, prognosis, nomogram model, genomic landscape

## Abstract

**Objective:**

The prognosis of breast cancer liver metastasis (BCLM) is poor, and its molecular mechanism is unclear. We aimed to determine the factors that affect the prognosis of patients with BCLM and investigate the genomic landscape of liver metastasis (LM).

**Methods:**

We described the prognosis of patients with BCLM and focused on prognosis prediction for these patients based on clinicopathological factors. Nomogram models were constructed for progression-free survival (PFS) and overall survival (OS) by using a cohort of 231 patients with BCLM who underwent treatment at Shandong Cancer Hospital and Institute (SCHI). We explored the molecular mechanism of LM and constructed driver genes, mutation signatures by using a targeted sequencing dataset of 217 samples of LM and 479 unpaired samples of primary breast cancer (pBC) from Memorial Sloan Kettering Cancer Center (MSKCC).

**Results:**

The median follow-up time for 231 patients with BCLM in the SCHI cohort was 46 months. The cumulative incidence of LM at 1, 2, and 5 years was 17.5%, 45.0%, and 86.8%, respectively. The median PFS and OS were 7 months (95% CI, 6–8) and 22 months (95% CI, 19–25), respectively. The independent factors that increased the progression risk of patients with LM were Karnofsky performance status (KPS) ≤ 80, TNBC subtype, grade III, increasing trend of CA153, and disease-free interval (DFS) ≤ 1 year. Simultaneously, the independent factors that increased the mortality risk of patients with LM were Ki-67 ≥ 30%, grade III, increasing trend of CA153, pain with initial LM, diabetes, and DFI ≤ 1 year. In the MSKCC dataset, the LM driver genes were ESR1, AKT1, ERBB2, and FGFR4, and LM matched three prominent mutation signatures: APOBEC cytidine deaminase, ultraviolet exposure, and defective DNA mismatch repair.

**Conclusion:**

This study systematically describes the survival prognosis and characteristics of LM from the clinicopathological factors to the genetic level. These results not only enable clinicians to assess the risk of disease progression in patients with BCLM to optimize treatment options, but also help us better understand the underlying mechanisms of tumor metastasis and evolution and provide new therapeutic targets with potential benefits for drug-resistant patients.

## Introduction

Breast cancer (BC) is a malignant tumor with the highest incidence in women, and the trend of rejuvenation is significant (1). Metastatic breast cancer (mBC) is constantly diagnosed, and the position of metastatic organs strongly correlates with survival time, despite progress has been made in the treatment and prognosis of early BC (2–4). The prognosis of liver recurrence is the second-worst outcome after brain metastasis (2, 4). About half of the patients with mBC eventually develop liver metastasis (LM) (5), and this probability is increasing every year (6).

The American Joint Committee on Cancer Staging is most commonly used to predict the prognosis of cancer patients. It includes TNM staging, pathological grade, and tumor expression status of biological indicators such as estrogen receptor (ER), progesterone receptor (PR), and human epidermal growth factor receptor 2 (HER-2). Additionally, some investigations have pointed out that biological and pathological parameters could be used for predicting the recurrence or prognosis of patients with BC (7, 8). However, an authoritative prediction model for the prognosis of patients with breast cancer liver metastasis (BCLM) has not yet been developed.

In the early years, the mechanisms of BCLM were not explored extensively, and mainly the inherent structure and microenvironment of the liver were focused on. For example, the high expression of Claudin-2 can enhance the adhesion to extracellular matrix proteins, thereby increasing the potential of BC cells to transfer to the liver (9). Another example is that the interaction between chemokine receptors on tumor cell membranes and chemokines in the microenvironment, such as CCL2 (10, 11) and CXCR4-CXCL12/SDF-1 (12–14), plays important roles in LM. Moreover, with the rapid development of sequencing technology in the recent years, a large number of early BC gene maps have been reported, along with several reports on the overall mutation characteristics of mBC (15–18). However, the genetic landscape related to LM alone has not yet been characterized.

**Figure 1:**
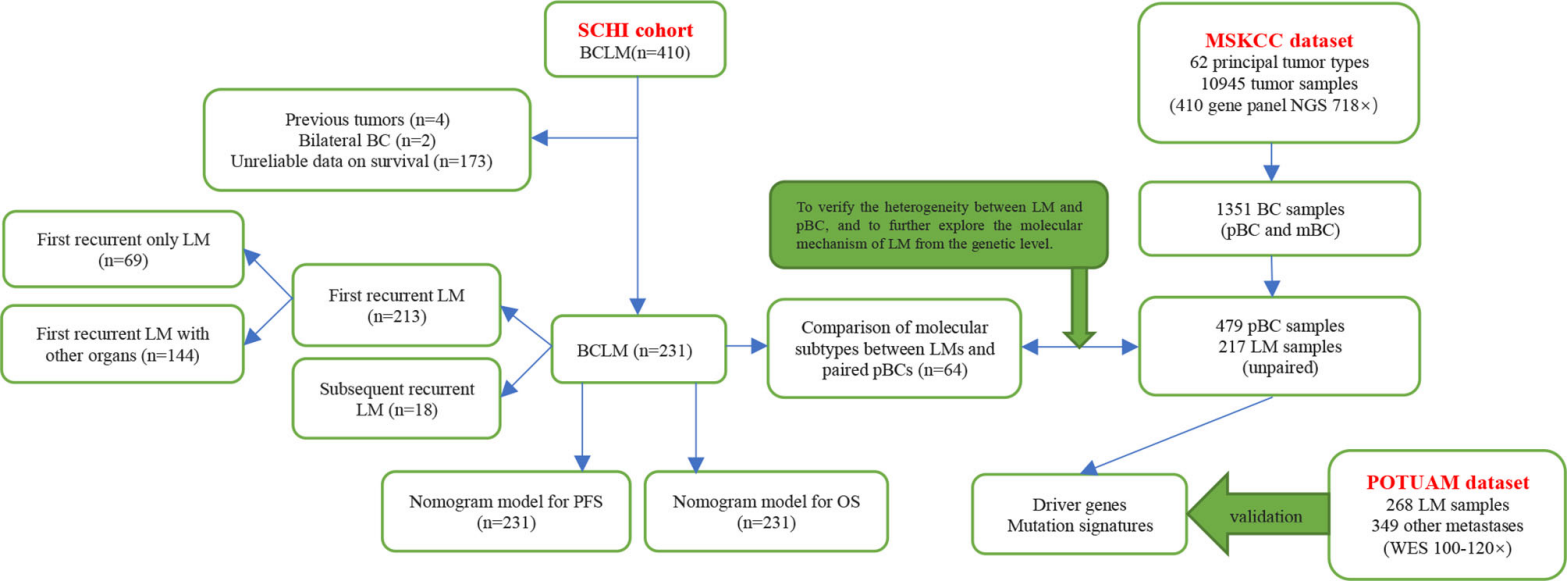
Article structure frame diagram. SCHI, Shandong Cancer Hospital and Institute; MSKCC, Memorial Sloan Kettering Cancer Center; POTUAM, Predictive Oncology team of the University of Aix-Marseille; NGS, next-generation sequencing; WES, whole-exome sequencing.

In this study, we aimed to focus on prognosis prediction and construct nomogram models for progression-free survival (PFS) and overall survival (OS) by using clinicopathological characteristics, especially the innovative application of the dynamic changes of hematological indicators, in a cohort of patients with BCLM who underwent treatment at Shandong Cancer Hospital and Institute (SCHI). We investigated the molecular mechanism of LM and constructed driver genes, mutation signatures (MSs) by using a targeted sequencing dataset from the Memorial Sloan Kettering Cancer Center (MSKCC), which was verified with the dataset of the Predictive Oncology team of the University of Aix-Marseille (POTUAM) (**Figure 1**).

## Materials and Methods

### SCHI Cohort

Patients who met the following criteria were eligible for this study: (1) diagnosed with BCLM and treated continuously with SCHI (**Figure 1**) from December 2008 to December 2018, (2) complete clinical pathology records, (3) age ≥ 18 and ≤ 75, and (4) Karnofsky performance status (KPS) ≥ 60. Patients who met any of the following criteria were excluded from the study: (1) bilateral BC, (2) primary and/or metastatic liver cancer, (3) other invasive malignant diseases within five years, and (4) medical records deemed unqualified according to the investigator’s opinion. All the medical records of patients with BCLM diagnosed by pathology or imaging were collected retrospectively. This study was approved by the Institutional Review Board, and the personal information of all patients and attending doctors was also deleted from the data set. Clinical features, pathological features, imaging examinations, treatment methods, and survival information were collected by two independent researchers according to a standardized process, and disagreements were resolved through discussions with a third expert.

The date of LM diagnosis was based on the date of the imaging examination or the date of biopsy pathology report. Disease-free interval (DFI) refers to the length of time from BC diagnosis to relapse or metastasis. PFS refers to the length of time from the diagnosis of BCLM to disease progression. OS refers to the length of time from BCLM diagnosis to death from any cause or the last follow-up. All surviving patients at the time of analysis were censored at the date of their last follow-up.

### MSKCC Dataset and POTUAM Dataset

The targeted sequencing dataset of 217 samples of LM and 479 unpaired samples of primary breast cancer (pBC) *via* MSK-IMPACT, a hybridization capture which has the ability to detect all protein-coding mutations, structural rearrangements, selected promoter mutations and copy number alterations in 410 cancer-associated genes on Illumina HiSeq sequencers, was extracted from the MSK-IMPACT Clinical Sequencing Cohort (19) (**Figure 1**) (https://www.cbioportal.org/study/summary?id=msk_impact_2017). MSK-IMPACT gene panels can be found here: https://github.com/cBioPortal/datahub/tree/master/reference_data/gene_panels. The POTUAM dataset contains the whole-exome sequencing results of 268 LM samples and 349 other metastasis samples (15) (**Figure 1**) (https://github.com/gustaveroussy/mBC_WES_Fabrice_Andre_2019).

### Significant Mutant Genes and Mutational Signatures

Significantly mutant genes were identified with *Maftools* (20) across the entire cohort of pBCs and LMs. *Oncodrive* based on the algorithm *oncodriveCLUST* (21) was used to identify cancer genes (driver) from a given mutant allele fractions. We then used *the NMF* (22) algorithm to decompose MSs based on the set of known signatures from the COSMIC database (23), and calculated the cosine similarity to identify the best match.

### Statistical Analysis

Chi-square test or Fisher’s exact test was used to evaluate differences in count data. Comparisons of tumor mutational burden were performed using Mann–Whitney U test. We used the Kaplan–Meier method for survival analysis and evaluated the difference between the Kaplan–Meier curves by applying the log-rank test. p < 0.05 was considered statistically significant. The Cox proportional hazards model was used to analyze the univariate and multivariate factors associated with survival. Finally, we used the *RMS* r package to draw the nomograms. All statistical analyses were performed using SPSS software for mac v26 and R v4.0.0.

## Results

### Clinicopathological Characteristics of the SCHI Cohort

Between December 2008 and December 2018, 410 patients with BCLM were admitted to our hospital. A total of 231 patients were eligible. Of the 231 patients, 213 had first recurrent LM and 18 had subsequent recurrent LM. Among the patients with first recurrent LM, 69 had only LM, and 144 had metastasis to other organs. (**Figure 1**). The median follow-up time in this study was 46 months (range, 12–118 months). The clinicopathological characteristics of the patients at baseline are shown in **Table 1**. The median age at diagnosis of mBC was 45 years (range: 26–72 years), of which 76 (32.9%) patients were ≤ 40 years old and 155 (67.1%) were menopausal. The most common anatomical location of the breast lump was the outer upper quadrant (39.4%), and the most common molecular subtype was HER-2 (33.8%). The number of patients in grades I-II and grade III was approximately equal (51.9% vs. 48.1%). A total of 183 patients (79.2%) with non-metastatic pBC eventually developed metastatic disease, while 48 (20.8%) presented with stage IV mBC at initial diagnosis. When diagnosed with mBC, 86 (37.2%) patients had 1 metastatic lesion, 82 (35.5%) had 2 metastatic lesions, and 63 (27.3%) had 3 or more metastatic lesions. The most commonly involved sites, apart from the liver, were the bone (n = 108), lung (n = 51), and brain (n = 9). During the course of the disease, 78 patients (33.8%) experienced clinical symptoms caused by LM. The most common symptoms of LM were abdominal pain (33.8%), vomiting (5.2%), and jaundice (2.6%). The median time from the diagnosis of BC to LM was 23 months [95% confidence interval (CI), 20–25]. The cumulative incidence of LM at 1, 2, and 5 years was 17.5%, 45.0%, and 86.8%, respectively.

**Table 1:** Clinicopathological characteristics of patients (N = 231).

Characteristics	No.	Percentage (%)
Age, years		
Median (Range)	45 (26–72)	
≤40	76	32.9
>40	155	67.1
Menopausal status		
Pre- or perimenopause	155	67.1
Post menopause	76	32.9
Location of breast lump		
Outside up	91	39.4
Outside down	36	15.6
Inside down	45	19.5
Inside up	54	23.4
Unknown	5	2.2
Subtypes		
Luminal A	56	24.2
Luminal B	63	27.3
Her-2	78	33.8
TNBC	34	14.7
Grade		
I-II	120	51.9
III	111	48.1
Neo/adjuvant therapy		
Anthracycline combined with paclitaxel	144	62.2
Others	87	37.7
Number of initial metastatic organs		
≤2	168	72.7
>2	63	27.3
Liver biopsy		
Yes	64	27.7
No	167	72.3
Initial site of mBC		
Liver	213	92.2
Bone	108	46.8
Lung	51	22.1
Brain	9	3.9
Local recurrence	36	15.6
With hepatitis		
Yes	9	3.9
No	222	96.1
DFI		
≤12m	36	15.6
>12m	153	66.2
IV	42	18.2
Liver metastasis within 1 year		
Yes	33	17.5
No	156	82.5
Liver metastasis within 2 year		
Yes	85	45.0
No	104	55.0

### Survival Following LM and Prognostic Factors

**Figure 2:**
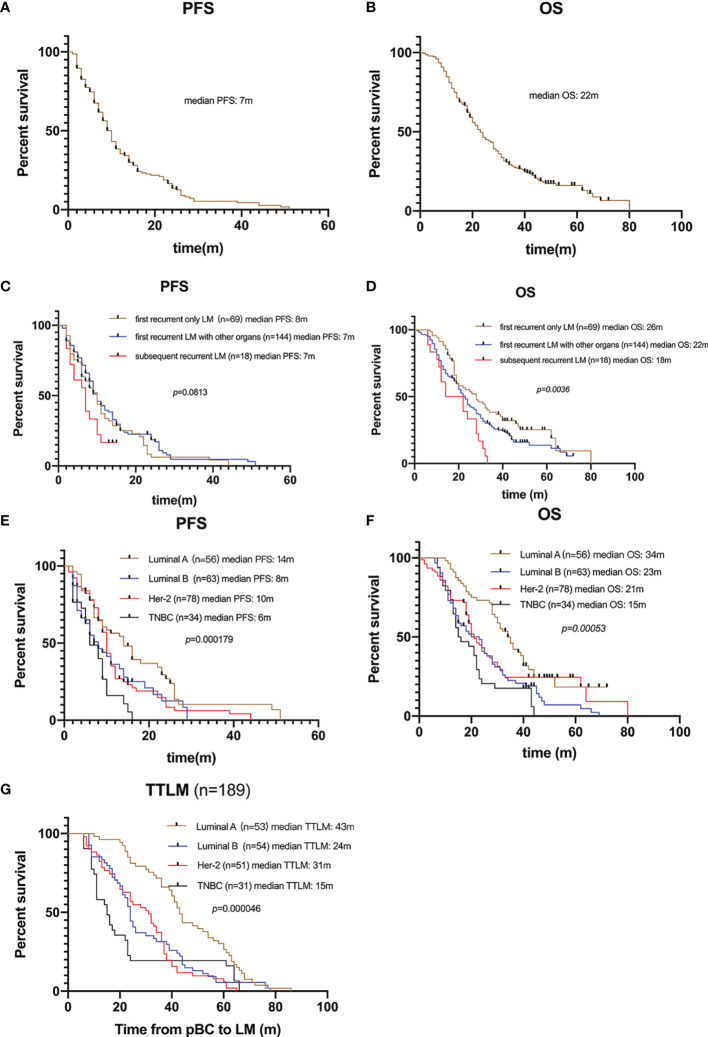
Survival of patients with LM. **(A)** Kaplan–Meier PFS curve of 231 patients with LM. **(B)** Kaplan–Meier OS curve of 231 patients with LM. **(C)** Kaplan-Meier plots illustrating PFS of patients with “only LM initially”, “LM with other organs initially”, and “subsequently recurrent LM”, respectively. No significant difference in PFS among groups (8 vs. 7 vs. 7 months, p = 0.0813). **(D)** Kaplan-Meier plots illustrating OS of patients with “only LM initially”, “LM with other organs initially”, and “subsequently recurrent LM”, respectively. Patients with only LM initial presence were associated with longer OS (26 vs. 22 vs. 18 months, p = 0.0036). **(E)** Kaplan-Meier plots illustrating PFS of patients with different molecular subtypes. Patients with TNBC were associated with shorter PFS (14 vs. 8 vs. 10 vs. 6 months, p = 0.000179). **(F)** Kaplan-Meier plots illustrating OS of patients with different molecular subtypes. Patients with TNBC were associated with shorter OS (34 vs. 23 vs. 21 vs. 15 months, p = 0.00053). **(G)** Kaplan-Meier plots illustrating time from pBC to LM (TTLM) of patients with different molecular subtypes. Patients with TNBC were associated with shorter TTLM (43 vs. 24 vs. 31 vs. 15 months, p = 0.00005).

One hundred and eighty-nine (81.8%) patients died by the end of the follow-up. For the 231 patients with BCLM in the SCHI cohort, the median PFS and OS were 7 months (95% CI, 6–8) and 22 months (95% CI, 19–25), respectively (**Figures 2A, B**). PFS at 1 year was 25.1%, and the OS at 1 and 2 years was 77.1% and 45.8%, respectively. Median PFS for patients with “first recurrent only LM” was longer than that for patients with “first recurrent LM with other organs” and “subsequent recurrent LM,” even no statistical difference was found (8 m 95% CI, 6–10 vs. 7 m 95% CI, 6–8 vs. 7 m 95% CI, 3.5–10, p = 0.0813). Meanwhile, the median OS for patients with “first recurrent only LM” was longer than that for patients with “subsequent recurrent LM with other organs” and “subsequent recurrent LM” (26 m 95% CI 19–33 vs. 22 m 95% CI 19–25 vs. 18 m 95% CI 11.5–28, p = 0.0036) (**Figures 2C, D**). Compared with other molecular subtypes, patients with TNBC were associated with shorter PFS (14 vs. 8 vs. 10 vs. 6 months, p = 0.000179) and shorter OS (34 vs. 23 vs. 21 vs. 15 months, p = 0.00053), and were more prone to LM at an early stage (43 vs. 24 vs. 31 vs. 15 months, p = 0.000046) (**Figures 2E–G**).

**Figure 3:**
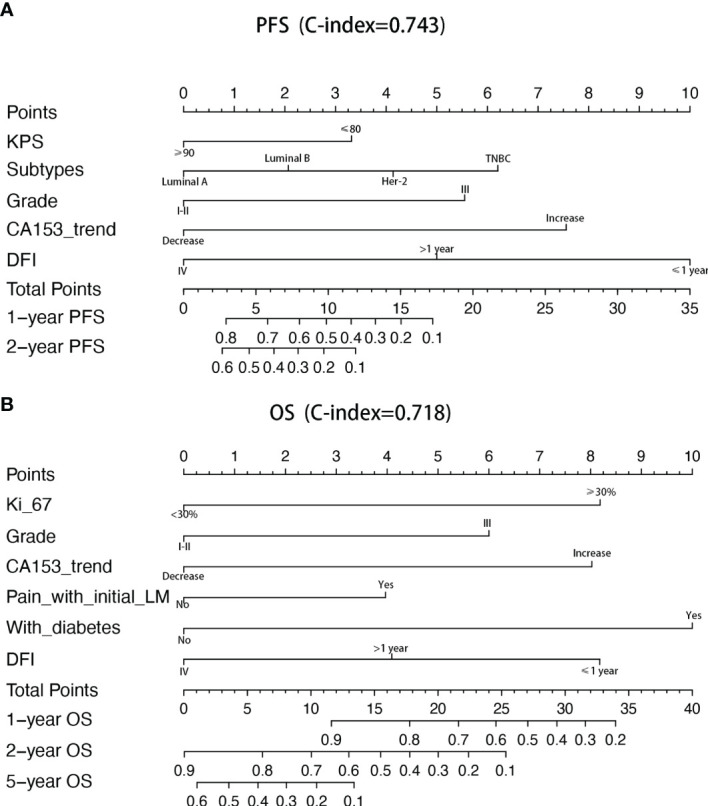
Nomograms of prognosis for patients with LM. **(A)** The nomogram of prognosis for patients with PFS (C-index = 0.743). **(B)** The nomogram of prognosis for patients with OS (C-index = 0.718).

Univariate and multivariate factors associated with survival were identified using the Cox proportional hazards model. Prognostic monogram models were established (**Figure 3**). The independent factors that increased progression risk of patients with LM were KPS ≤ 80 [hazard ratio (HR): 1.68, 95% CI: 1. 15–2.45; p = 0.007], TNBC subtype (HR: 2.61, 95% CI: 1.35–5.04; p = 0.038), grade III (HR: 2.09, 95% CI: 1. 48–2.96; p < 0.001), increasing trend of CA153 (HR: 2.79, 95% CI: 1. 86–4.16; p < 0.001), and DFI ≤ 1 year (HR: 4.09, 95% CI: 2.37–7.07; p < 0.001) (**Table 2**, **Figure 3A**). Simultaneously, the independent factors that increased death risk of patients with LM were Ki-67 ≥ 30% (HR: 2.74, 95% CI: 1.45–5.02; p = 0.001), grade III (HR: 2.19, 95% CI: 1. 53–3.14; p < 0.001), increasing trend of CA153 (HR: 2.32, 95% CI: 1. 60–3.37; p < 0.001), pain with initial LM (HR: 2.02, 95% CI: 1.18–3.44; p = 0.010), diabetes (HR: 4.47, 95% CI: 2.48–8.08; p < 0.001), and DFI ≤ 1 year (HR: 2.42, 95% CI: 1.32–4.43; p = 0.011) (**Table 3**, **Figure 3B**).

**Table 2:** Univariate and multivariate models for PFS with BCLM patients (n = 231).

Factor	No.	Univariate		p	Multivariate		p
		Median	95% CI		HR	95% CI	
Age				0.060			
≤40	76	10	8.8–11.1				
>40	155	9	6.7–11.2				
Menopausal status				0.535			
Pre- or perimenopause	155	10	8.9–11.0				
Post menopause	76	8	5.9–10.0				
Neo/adjuvant therapy				0.271			
Anthracycline combined with paclitaxel	144	10	7.4–12.5				
Others	87	9	7.0–8.9				
Number of initial metastatic organs				0.140			
≤2	168	10	8.6–11.3				
>2	63	7	5.3–8.6				
Bilirubin				0.129			
Normal	189	9	8.0–9.9				
Above limit	42	11	1.0–20.9				
G/L trend∗				0.051			
Decrease	101	10	8.5–11.4				
Increase	106	8	6.6–9.3				
With hepatitis				0.329			
Yes	9	8	5.6–10.4				
No	222	10	8.7–11.2				
Pain with initial LM				0.115			
Yes	31	8	0.0–16.3				
No	200	10	9.0–10.9				
Diabetes				0.973			
Yes	19	13	7.2–18.7				
No	212	9	7.9–10.0				
KPS				0.001			0.007
≥90	180	10	9.0–10.9				
≤80	51	6	3.5–8.4		1.68	1.15–2.45	
Ki-67				0.000			0.221
<30%	108	13	10.3–15.6				
≥30%	123	8	6.7–9.2		1.36	0.83–2.22	
Subtypes				0.000			0.038
Luminal a	56	14	9.9–18.0				
Luminal b	63	8	4.4–11.5		1.73	0.89–3.36	0.106
Her-2	78	10	9.0–10.9		1.50	0.90–2.48	0.113
TNBC	34	6	3.7–8.2		2.61	1.35–5.04	0.004
Grade				0.000			0.000
I-II	120	12	9.4–14.5				
III	111	9	7.4–10.5		2.09	1.48–2.96	
Number Of LM				0.007			0.331
Single	51	14	9.7–18.2				
Multiple	180	8	6.7–9.2		1.23	0.80–1.90	
CA153 trend*				0.000			0.000
Decrease	141	11	9.1–12.8				
Increase	90	6	4.8–7.1		2.79	1.86–4.16	
CEA trend^#^				0.001			0.614
Decrease	144	10	8.8–11.1				
Increase	87	6	3.9–8.0		1.09	0.76–1.56	
DFI				0.000			0.000
≤1 year	36	5	3.4–6.5		4.09	2.37–7.07	0.000
>1 year	153	10	6.7–13.2		1.52	0.97–2.37	0.064
IV	42	11	9.4–12.5				

^*＊#^: After 2 cycles of treatment, the changing trend of Granulocyte/Lymphocyte, CA153 and CEA.

**Table 3:** Univariate and multivariate models for OS with BCLM patients (n = 231).

Factor	No.	Univariate		p	Multivariate		p
		Median	95%CI		HR	95%CI	
Age				0.619			
≤40	76	20	14.6–25.3				
>40	155	24	19.0–26.9				
Menopausal status				0.994			
Pre- or perimenopause	155	24	20.2–27.7				
Post menopause	76	20	16.7–23.2				
Neo/adjuvant therapy				0.676			
Anthracycline combined with paclitaxel	144	24	20.2–27.7				
Others	87	21	14.9–27.0				
Number of LM				0.071			
Single	51	31	23.1–38.8				
Multiple	180	22	19.7–24.2				
Bilirubin				0.057			
Normal	189	23	20.4–25.5				
Above limit	42	21	10.4–31.5				
CEA trend^#^				0.084			
Decrease	144	24	20.7–27.2				
Increase	87	20	15.2–24.7				
With hepatitis				0.121			
Yes	9	24	22.6–25.3				
No	222	23	18.8–27.1				
KPS				0.001			0.081
≥90	180	24	20.1–27.8				
≤80	51	19	14.3–23.6		1.43	0.95–2.13	
Ki-67				0.000			0.001
<30%	108	33	28.8–37.1				
≥30%	123	19	16.9–21.0		2.74	1.49–5.02	
Subtypes				0.001			0.496
Luminal a	56	34	39.0–38.9				
Luminal b	63	23	15.7–20.2		1.06	0.50–2.26	0.868
Her-2	78	21	17.6–24.3		1.23	0.70–2.14	0.460
TNBC	34	15	7.8–22.1		1.61	0.76–3.40	0.212
Grade				0.000			0.000
I-II	120	28	23.4–32.5				
III	111	20	17.3–22.6		2.19	1.53–3.14	
Number of initial metastatic organs				0.000			0.964
≤2	168	28	24.4–31.5				
>2	63	11	8.4–13.5		1.01	0.63-1.60	
CA153 trend∗				0.000			0.000
Decrease	141	29	25.7–32.2				
Increase	90	16	13.2–18.7		2.32	1.60–3.37	
G/L trend^*^				0.048			0.149
Decrease	101	28	23.2–32.7				
Increase	106	23	20.9–25.1		1.29	0.91–1.83	
Pain with initial LM				0.029			0.010
Yes	31	19	17.1–20.8		2.02	1.18–3.44	
No	200	24	21.0–26.9				
Diabetes				0.001			0.000
Yes	19	15	12.8–17.1		4.47	2.48–8.08	
No	212	24	20.9–27.0				
DFI				0.000			0.011
≤1 year	36	15	7.6–22.3		2.42	1.32–4.43	0.004
>1 year	153	24	18.0–29.9		1.18	0.75–1.85	0.469
IV	42	25	21.4–28.5				

^#＊*^: After 2 cycles of treatment, the changing trend of CEA, CA153 and Granulocyte/Lymphocyte.

### Genetic Changes During BCLM

**Figure 4:**
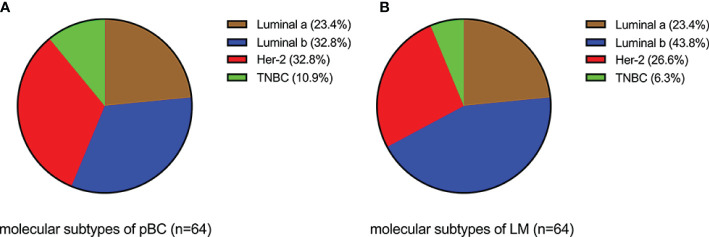
Comparison of molecular subtypes between LMs and paired pBCs (p = 1.92 × 10^−11^). **(A)** Components of molecular subtypes of pBC. **(B)** Components of molecular subtypes of LM.

**Figure 5:**
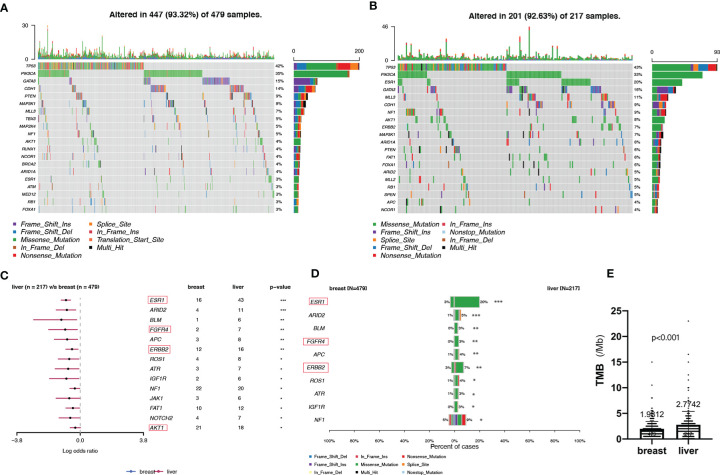
Somatic mutations in pBCs and LMs. **(A, B)** Top 20 SMGs in pBCs and LMs, respectively. **(C)** Comparison of SMGs between pBCs and LMs. **(D)** Comparison of the top 10 SMGs between pBCs and LMs. (The genes in the red box represent the driver genes, *p < 0.05, **p < 0.01, ***p < 0.001) **(E)** Comparison of TMB between pBCs and LMs (p < 0.001). SMG, significantly mutanted gene; TMB, Tumor Mutation Burden.

Analysis of the immunohistochemical status of 64 patients with LM and paired pBC in the SCHI cohort showed that the molecular subtype mutation rate of LM was 32.8% (21/64) compared with paired pBC. (Fisher’s exact test, p = 1.92 × 10^−11^) (**Figure 4**). This indicates that breast cancer cells have mutated during metastasis to the liver. To verify the heterogeneity between LM and pBC, and to further explore the somatic changes in patients with BCLM, we re-analyzed the targeted sequencing results of the MSKCC dataset, which contains the sequencing data of 217 samples of LM and 479 samples of pBC extracted from the MSK-IMPACT Clinical Sequencing Cohort (19). Overall, we used the maftools R package to identify the top 10 significantly mutanted genes (SMGs) in LM: TP53 (43%), PIK3CA (33%), ESR1 (20%), GATA3 (16%), MLL3 (11%), CDH1 (9%), NF1 (9%), AKT1 (8%), ERBB2 (7%), and MAP3K1 (7%) (**Figure 5B**). Compared with the mutated genes identified in the pBC (**Figure 5A**), ESR1, ARID2, BLM, FGFR4, APC, ERBB2, ROS1, ATR, IGF1R, NF1, JAK1, FAT1, NOTCH2, and AKT1 mutation frequencies were significantly different in LM. Among them, the driver genes were ESR1 (20%), AKT1 (8%), ERBB2 (7%), and FGFR4 (4%) (**Figures 5C, D**). Additionally, their mutation frequencies among the LM samples in the POTUAM dataset were 24% (ESR1), 7% (AKT1), 6% (ERBB2), and 1% (FGFR4) (**Supplementary Figure 1**).

**Figure 6:**
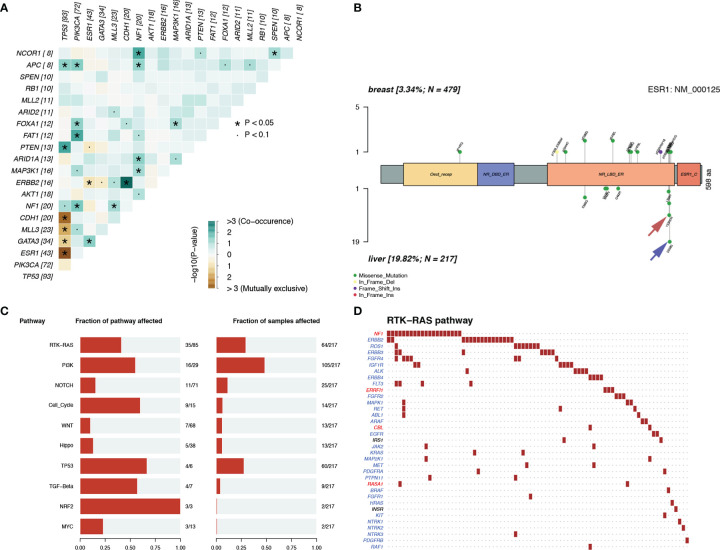
Mutation patterns in patients with LM. **(A)** Mutually exclusive or co-occurring set of genes. **(B)** Lolliplot of ESR1 mutation rate in the patients with pBC and LM. **(C, D)** Enrichment of known Oncogenic Signaling Pathways (Tumor suppressor genes are in red, and oncogenes are in blue font).

Notably, we observed highly accumulated ESR1 mutations in LM, and the ESR1 mutation rate of LM (20%) was significantly higher than that of pBC (3%) (Fisher’s exact test, p = 6.20 × 10^−12^) (**Figure 5D**). TP53, as the gene with the highest mutation rate, mutated exclusively with ESR1 in LM (pair-wise Fisher’s exact test, p = 6.11 × 10^−5^) (**Figures 5B**, **6A**). ERBB2, which also had a high mutation frequency in LM, was another gene that was mutually exclusive with the ESR1 mutation (**Figures 5B**, **4A**). Moreover, we found that almost all GATA3 mutations were accompanied by ESR1 mutations (**Figure 6A**), and the incidence of GATA3 mutations in LM was slightly higher than that in pBC. ESR1 mutations in LM patients were not only more frequent but also more concentrated in mutation sites, in contrast to the low frequency and scattered mutation patterns in patients with pBC. Thirty-five LM samples in total carried mutations were resistant to ESR1 aromatase inhibitor (AI) (D538G:19, Y537S/N:16), compared with only three pBC samples (D538G:1, Y537S/N:2) (Fisher’s exact test, p = 8.37 × 10^−16^) (**Figure 6B**).

To discover the biological pathways that play a key role in LM, we enriched the mutation matrix with known oncogenic signaling pathways in TCGA cohorts (24). Four oncogenes (FGFR4, ERBB2, ROS1, and IGF1R) and one suppressor gene (NF1) in the RTK-RAS pathway, which ranked first, were mutated more frequently in LM (**Figures 6C, D**).

### Three MSs in Patients With BCLM

**Figure 7:**
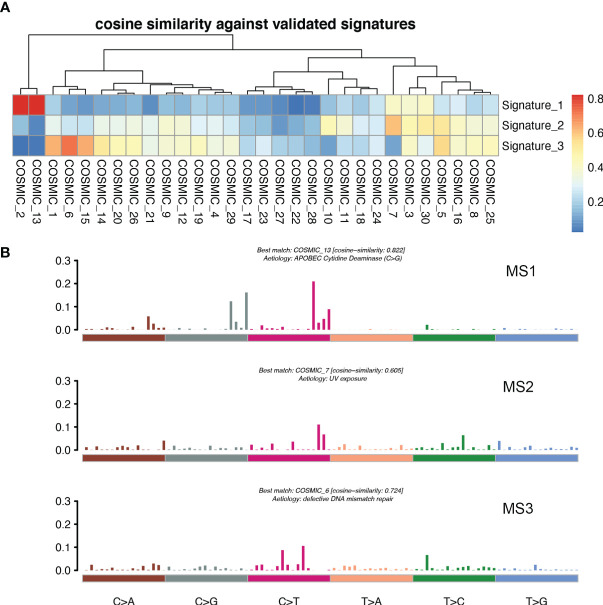
MSs in patients with LM. **(A)** Correlation between the MSs derived from the patients with LM and previously defined signatures from COSMIC. A pair-wise cosine correlation was performed between the COSMIC and LM signatures. The most correlated COSMIC signatures were used to determine the identity of each LM signature. **(B)** Three MSs identified in patients with LM.

For the purpose of clarifying the etiological mechanism of the different mutation rates between LM and pBC in the MSKCC dataset, and explaining the potential mechanism of BC metastasis to the liver, non-negative matrix factorization (NMF) created by Nik-Zainal S et al. (22) was used to extract MSs from 96 subtypes of three-base context of mutations. A total of three prominent signatures were matched (**Figure 7A**): MS1, best matched to COSMIC signature 13 (cosine-similarity: 0.822), is characterized by C > T mutations at the TpC dinucleotide and an APOBEC Cytidine Deaminase (C > G) phenotype; MS2, best matched to COSMIC signature 7 (cosine-similarity: 0.605), is characterized mainly by C > T and T > C mutations with other types of base substitutions contributing less and intricate patterns formed due to exposure to ultraviolet; and MS3, best matched to COSMIC signature 6 (cosine-similarity: 0.724), is mainly characterized by C > T mutations and a map caused by defective DNA mismatch repair (**Figure 7B**).

## Discussion

We described the prognosis of patients with BCLM, established a prognostic prediction model based on clinical pathological factors, and characterized the genomic landscape of patients with LM with external dataset in this study. We innovatively incorporated the dynamic changes of traditional tumor markers, such as CEA, CA153, and blood indicators, such as granulocyte-lymphocyte ratio, in the early stage of treatment into the prognostic analysis of LM. It is encouraging to note that the dynamic changes of CA153 greatly improved the prediction performance of the model.

The median follow-up time for the 231 patients with BCLM in the SCHI cohort was 46 months. In this cohort, the cumulative incidence of LM within 5 years was as high as 86.8%. The median PFS and OS were 7 months (95% CI, 6–8) and 22 months (95% CI, 19–25), respectively (**Figures 2A, B**). PFS at 1 year was 25.1%, and the OS at 1 and 2 years was 77.1% and 45.8%, respectively. We found that the OS of patients with first recurrent only LM (n = 69) was significantly longer than that of patients with first recurrent LM with other organs (n = 144) and subsequent recurrent LM (n = 18) (p = 0.0036). This may be because patients with LM as the only first site of metastasis had better KPS (chi-square test, p = 0.005). Conversely, patients who are accompanied by metastasis to other organs tend to have heavier tumor burden and higher tumor heterogeneity, and are more susceptible to drug resistance. In addition, our results support the view that the natural process of LM is also strongly influenced by the biology of BC subtypes, which is consistent with previous studies (6, 25, 26). Patients with TNBC subtype were more prone to LM in the early postoperative period (**Figure 2G**) and had shorter PFS (**Figure 2E**) and OS (**Figure 2F**). Considering that the molecular subtypes of 64 LMs obtained a 32.8% (21/64) mutation rate compared with the paired pBCs (**Figure 4**), biopsies of metastasis are useful for the reassessment of the metastatic sites to define a more effective treatment strategy for patients with BCLM (27). Thus, the ER, PR, and HER-2 statuses need to be reassessed by biopsy when LM occurs. The indicators of KPS, Ki-67, and grade were recorded in previous reports of mBC. KPS ≤ 70 (28), Ki-67 ≥ 20% (29, 30), and grade III (31) were independent risk factors for the prognosis of mBC. These results are generally consistent with those of our study. Moreover, in a study by Nishimura et al. (29), DFI is inversely correlated with the Ki-67 values. We also observed that the shorter the DFI, the worse the prognosis of patients with LM. Finally, our data showed, for the first time, that patients with diabetes and initial pain in the liver have a shorter OS.

In addition to the above factors that have an impact on the prognosis of LM, we have included the dynamic changes of CA153, CEA, and granulocyte-lymphocyte ratio after two cycles of LM treatment as prognostic factors into consideration for the first time. The increasing trend of CA153 showed a strong correlation with shorter PFS (HR: 2.79, 95% CI: 1. 86–4.16; p < 0.001) and OS (HR: 2.32, 95% CI: 1. 60–3.37; p < 0.001). More importantly, the dynamic changes of CA153 could reflect the curative effect in a timelier and more accurate manner, and help decision makers adjust the treatment plan according to the curative effect. To summarize, we established a prognostic model for BCLM based on these clinical and pathological factors (**Figure 3**). Compared with the prognostic nomogram model without CA153_trend (**Supplementary Figure 2**), the prognostic nomogram model including the dynamic changes of CA153 had greatly improved predictive ability (C-index: 0.743 vs. 0.693 for PFS, 0.718 vs. 0.673 for OS). Aiding with this nomogram, clinicians might be able to assess the risk of disease progression in patients with BCLM to optimize treatment options and speculate the patient’s risk of death to avoid meaningless treatment.

In the SCHI cohort, the critical cause of the 32.8% molecular subtype mutation rate of LMs relative to pBCs is changes at the genome level. The genomic landscape of early BC has been reported many times. In addition, there is evidence that genomic changes are obtained in the process of cancer metastasis and progression and the genomic pattern of early cancers cannot represent lethal cancers (32–36). Recently, there have been subsequent reports on the genomic landscape of mBC (16–18). However, the molecular mechanism for LM remains an area that has not yet been fully developed (37). In view of the above, we decoded the genomic landscape of LM by using the MSKCC dataset (19). Among the 14 SMGs in the LM samples, we identified four driver genes: ESR1 (20%), AKT1 (8%), ERBB2 (7%), and FGFR4 (4%). Compared with the 21 potential driver genes (TP53, ESR1, CDH1, MAP3K1, GATA3, CBFB, ARID1A, ERBB2, RUNX1, MAP2K4, GPS2, FOXA1, TBX3, NCOR1, PTEN, PIK3CA, KMT2C, RB1, AKT1, CDKN1B, and NF1) of mBC in the study by Angus et al. (16), most of the four driver genes (3/4, ESR1, AKT1, and ERBB2) of LM were more consistent, verifying that ESR1, ERBB2, and AKT1 can drive LM. The other 18 inconsistent driver genes of mBC also indicate that the driver genes of LM are not exactly the same as those of other metastases, that is to say these inconsistent driver genes of mBC may play a role in metastasis in other organs, but not in liver. To further verify our results, we quoted the POTUAM dataset. The mutation frequencies of the four driver genes in the MSKCC dataset were highly consistent with those in the POTUAM dataset (**Supplementary Figure 1**). Furthermore, in the POTUAM dataset, except for FGFR4, which had a low mutation rate, the mutation frequencies of ESR1, AKT1, and ERBB2 in the LM were higher than those in other metastases. Moreover, LM had a significantly different mutation spectrum from other metastases (**Supplementary Figure 3**). Our study characterized the driver genes of LM isolated from other metastases, making the molecular targets of LM clearer and more focused.

Interestingly, ESR1 and TP53 were the most mutually exclusive mutant gene pair in LMs (pairwise Fisher’s exact test, p = 6.11 × 10^−5^). As previously reported (15, 38), ESR1 mutations were enriched in patients with LMs (Fisher’s exact test, P < 0.001). In contrast to the low frequency and scattered mutation patterns in the pBC cohort, the ESR1 mutations were mainly concentrated in D538G and Y537S/N. This is consistent with a previous study published in the journal JAMA Oncology (39). Another notable gene is NF1. In a recent study, Bertucci et al. (15) performed whole-exome sequencing of 617 tumor samples from patients with metastatic BC and revealed that the mutation frequency of NF1 is negatively correlated with the prognosis of patients with HR +/HER-2- mBC. In the current study, we found that as a tumor suppressor gene, NF1 had the most frequent mutation in the RTK-RAS pathway in patients with LM. To better understand which mutation processes facilitated the progression of LM, we further evaluated the distribution of MSs. LMs matched three prominent signatures: S13 (APOBEC cytidine deaminase), S7 (ultraviolet exposure), and S6 (defective DNA mismatch repair). According to previous reports, APOBEC activation can mediate secondary resistance to endocrine therapy (40). Evaluating BCLM using a next-generation sequencing approach helps us better understand the underlying mechanisms of tumor metastasis and evolution and provide new therapeutic targets with potential benefits for drug-resistant patients. Moreover, owing to the improvement of the current circulating tumor DNA detection technology, if the mutant LM driver genes can be detected in the circulating blood, it may be a powerful tool for early warning of LM. Finally, by comparing the TMB between LMs and pBCs (2.77 vs. 1.98, U test, p < 0.001), we also confirmed that metastatic foci are more genetically complex than primary foci, as in previous studies (32, 33, 35).

Our study has several limitations. First, this research was retrospective, so there may be some unavoidable biases. Second, the construction of the prognosis prediction model was based on a SCHI single-center cohort; thus a prospective multi-center verification is required before subsequent promotion. Third, we used an external sequencing dataset to characterize the genomic landscape of BCLM, and further exploration requires the support of more cell and animal experiments.

In summary, BCLM is a complex process that involves many factors. This study systematically describes the survival prognosis and the characteristics of LM from clinicopathological factors to the genetic level. The independent factors that increased the progression risk of patients with LM were KPS ≤ 80, TNBC subtype, grade III, increasing trend of CA153, and DFI ≤ 1 year. Simultaneously, the independent factors that increased the mortality risk of patients with LM were Ki-67 ≥ 30%, grade III, increasing trend of CA153, pain with initial LM, diabetes, and DFI ≤ 1 year. Mutations in the driver genes ESR1, AKT1, ERBB2, and FGFR4, and the MS APOBEC cytidine deaminase, ultraviolet exposure, and defective DNA mismatch repair may provide convenience for LM. We believe that our study makes a significant contribution to the literature because it can help clinicians evaluate the risk of disease progression in patients with BCLM to determine the optimal treatment strategy. Our results also provide a better understanding of the mechanisms underlying BCLM progression and evolution and provide new therapeutic targets that can potentially benefit drug-resistant patients or be eligible for clinical trials.

## Data Availability Statement

The original contributions presented in the study are included in the article/**Supplementary Material**. Further inquiries can be directed to the corresponding authors.

## Ethics Statement

The study was approved by the institutional review board of Shandong Cancer Hospital and Institute, and the personal information of all patients and attending doctors was deleted from the data set. The ethics committee waived the requirement of written informed consent for participation.

## Author Contributions

CT collected the patient’s clinicopathological data and downloaded external data sets. SL followed up the patient’s survival information. CT and SL jointly analyzed the data and prepared the manuscript. XS and YW supervised the research and revised the manuscript. All authors contributed to the article and approved the submitted version.

## Funding

This work was supported by the National Natural Science Foundation of China (81672104). The funder had no role in study design, data collection and analysis, decision to publish, or preparation of the manuscript.

## Conflict of Interest

The authors declare that the research was conducted in the absence of any commercial or financial relationships that could be construed as a potential conflict of interest.

## Supplementary Material

Supplementary Figure 1 Comparison of the mutation frequency of the four driver genes (**A**. ESR1 **B**. FGFR4 **C**. ERBB2 **D**. AKT1) between the MSKCC dataset and the POTUAM dataset. (chi-square test).

Supplementary Figure 2 Nomograms of prognosis for patients with LM (excluding CA153_trend). **(A)** The nomogram of prognosis for patients with PFS (C-index = 0.693). **(B)** The nomogram of prognosis for patients with OS (C-index = 0.673).

Supplementary Figure 3 Comparison of SMGs between liver metastases and other metastases in the POTUAM dataset. (SMG, significantly mutanted gene. *p < 0.05, **p < 0.01, ***p < 0.001).
