# Supplementary figures and images for: Prognosis and Genomic Landscape of Liver Metastasis in Patients With Breast Cancer
Source: Front Oncol. 2021 Mar 11;11:588136. doi: 10.3389/fonc.2021.588136 (PMC7991092; doi:10.3389/fonc.2021.588136)

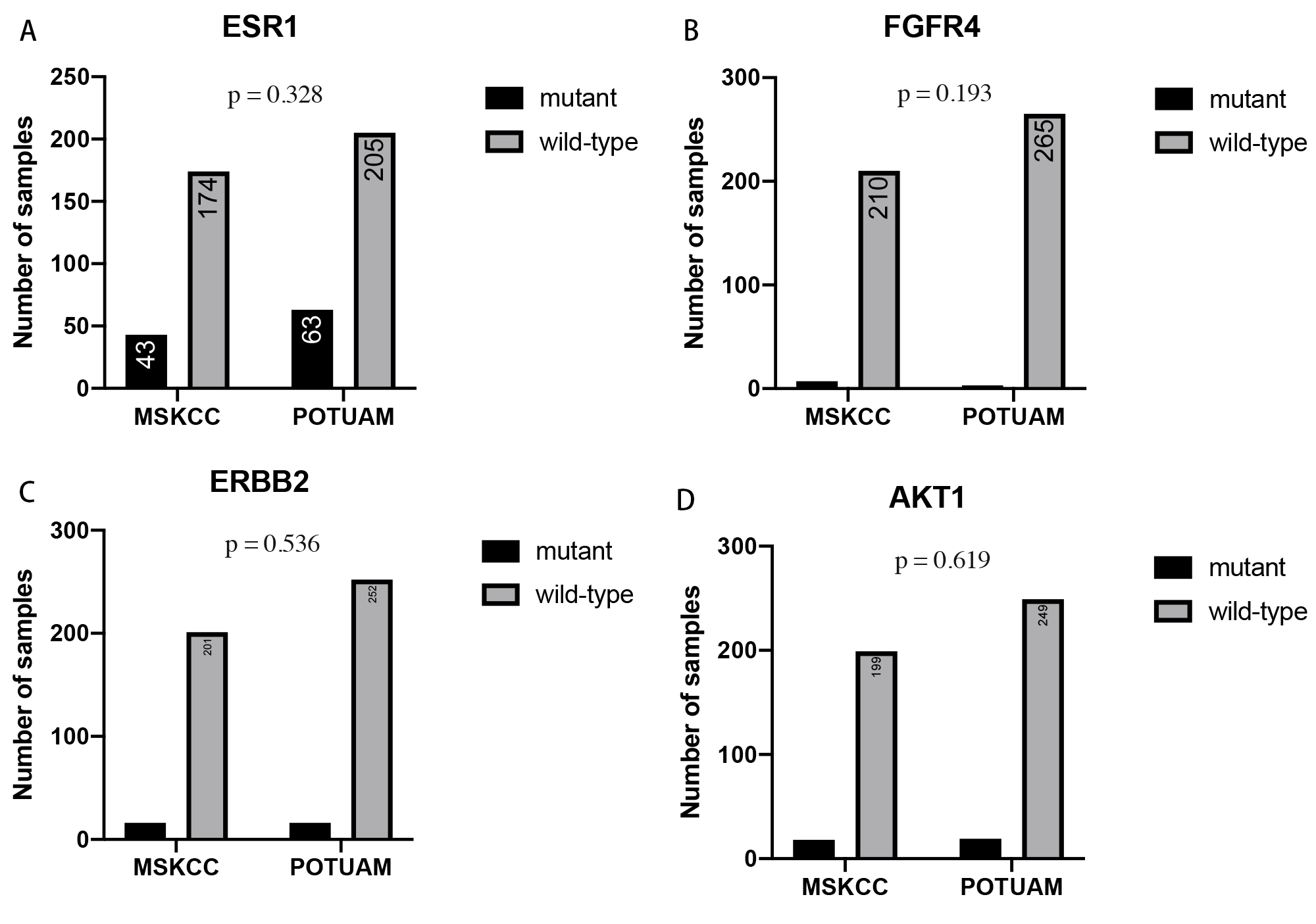

Supplement: Supplementary Figure 1 — Comparison of the mutation frequency of the four driver genes (A. ESR1 B. FGFR4 C. ERBB2 D. AKT1) between the MSKCC dataset and the POTUAM dataset. (chi-square test). [file Image_1.tif]

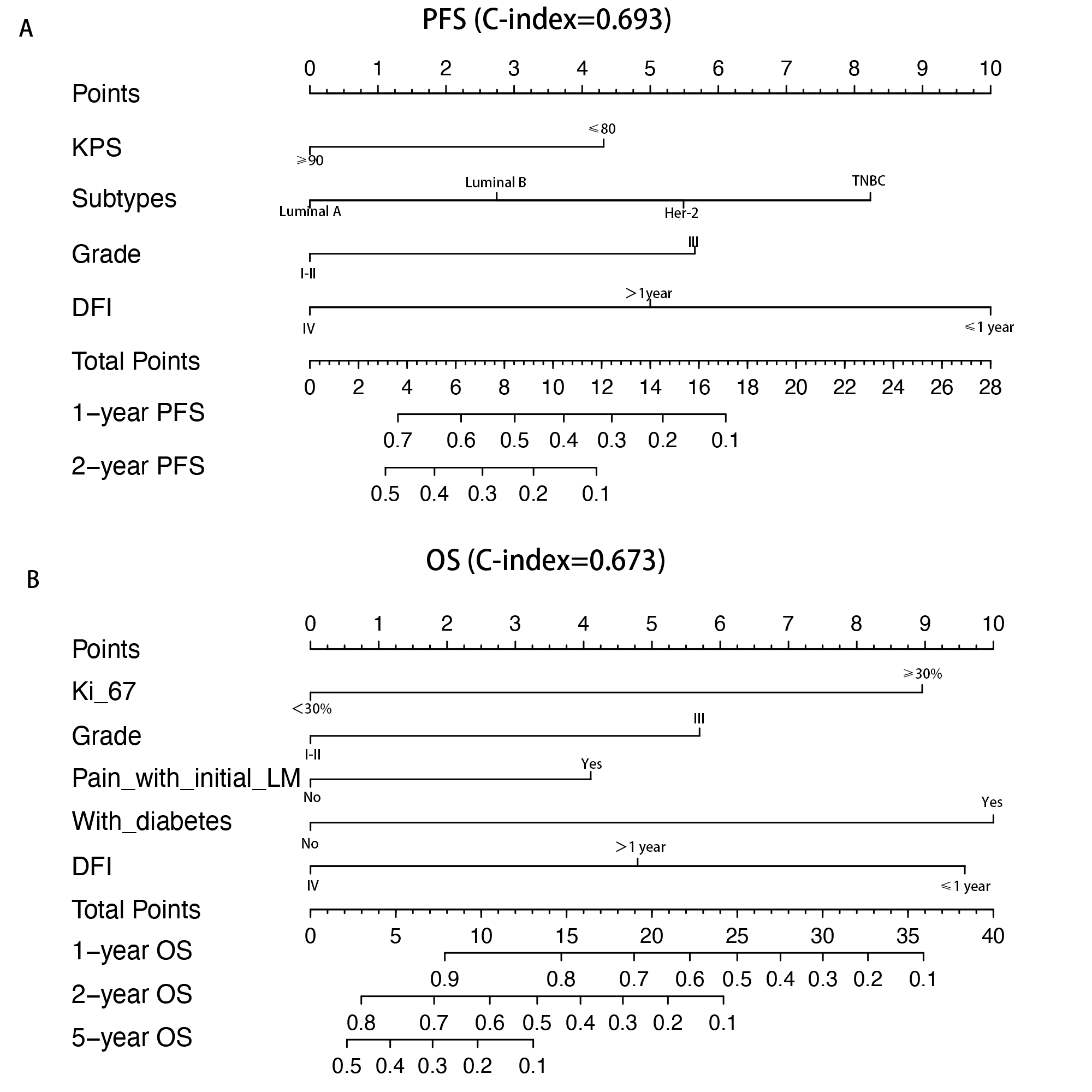

Supplement: Supplementary Figure 2 — Nomograms of prognosis for patients with LM (excluding CA153_trend). (A) The nomogram of prognosis for patients with PFS (C-index = 0.693). (B) The nomogram of prognosis for patients with OS (C-index = 0.673). [file Image_2.tif]

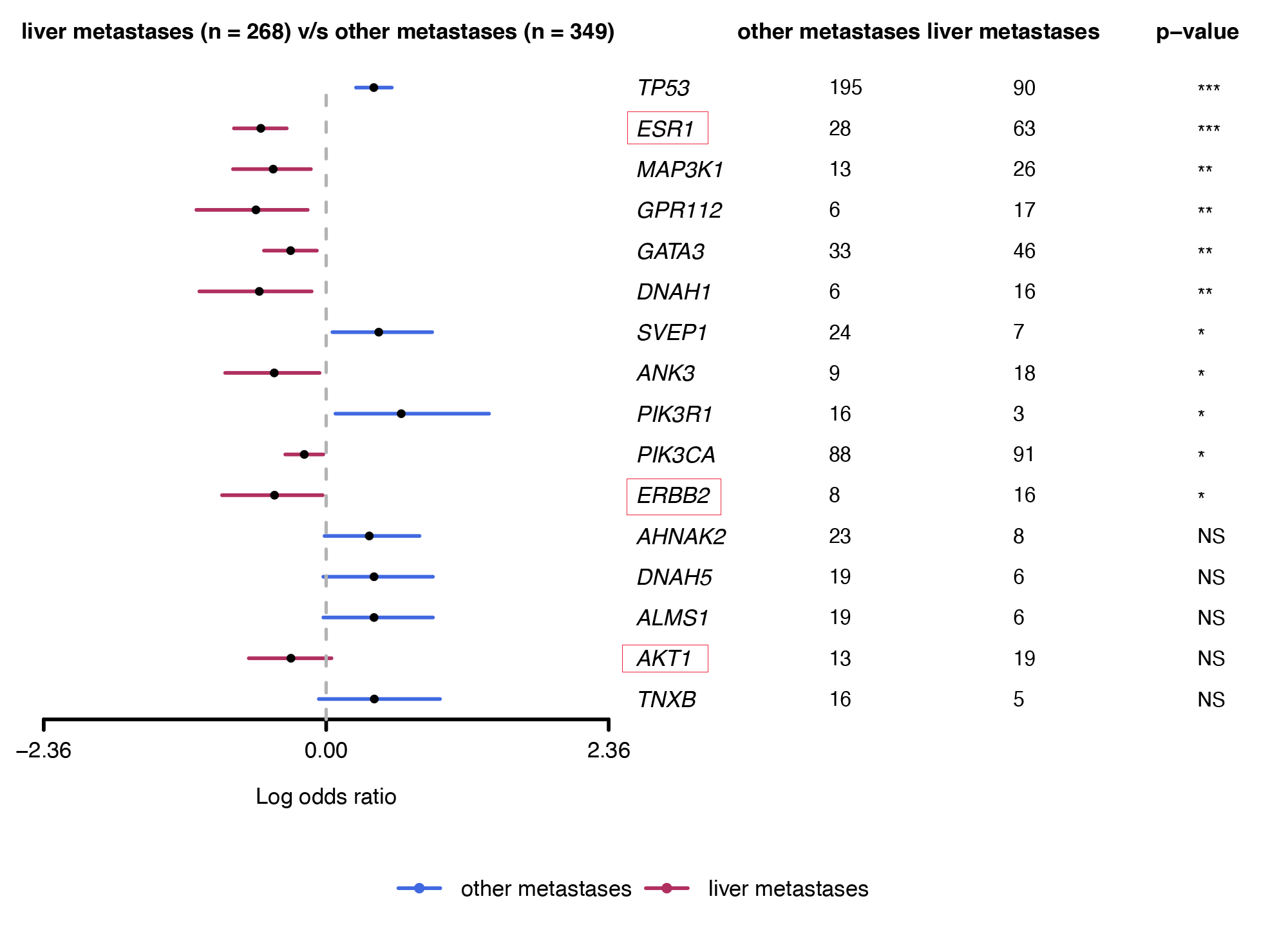

Supplement: Supplementary Figure 3 — Comparison of SMGs between liver metastases and other metastases in the POTUAM dataset. (SMG, significantly mutanted gene. *p < 0.05, **p < 0.01, ***p < 0.001). [file Image_3.tif]
